# Natural Neuroinflammatory Modulators: Therapeutic Potential of Fungi-Derived Compounds in Selected Neurodegenerative Diseases

**DOI:** 10.3390/molecules30153158

**Published:** 2025-07-28

**Authors:** Agnieszka Godela, Diana Rogacz, Barbara Pawłowska, Robert Biczak

**Affiliations:** The Faculty of Science and Technology, Jan Długosz University in Czestochowa, 13/15 Armii Krajowej Av., 42-200 Czestochowa, Poland; a.godela@ujd.edu.pl (A.G.); d.rogacz@ujd.edu.pl (D.R.); b.pawlowska@ujd.edu.pl (B.P.)

**Keywords:** fungi, natural nutraceuticals, neurodegenerative diseases, bioactive metabolites, neuroinflammation, neuroprotection

## Abstract

Neurodegenerative diseases such as Alzheimer’s disease, Parkinson’s disease, Huntington’s disease, and amyotrophic lateral sclerosis remain incurable. Current therapeutic strategies primarily focus on slowing disease progression, alleviating symptoms, and improving patients’ quality of life, including the management of comorbid conditions. Over the past few decades, the incidence of diagnosed neurodegenerative disorders has risen significantly. As the number of affected individuals continues to grow, so does the urgent need for effective treatments that can halt or mitigate the progression of these diseases. Among the most promising therapeutic resources are bioactive compounds derived from fungi. The high quality of proteins, polysaccharides, unsaturated fatty acids, triterpenoids, sterols, and secondary metabolites found in fungi have attracted growing interest from researchers across multiple disciplines. One intensively studied direction involves the use of naturally occurring fungi-derived nutraceuticals in the treatment of various diseases, including neurodegenerative conditions. This article provides an overview of recent findings on fungal compounds—such as phenolic compounds, carbohydrates, peptides and proteins, and lipids—that may have potential applications in the treatment of neurodegenerative diseases and the alleviation of their symptoms.

## 1. Introduction

Neurodegenerative disorders (NDDs) represent a significant global health challenge, ranking among the leading causes of disability and morbidity worldwide. Their increasing prevalence—particularly in aging populations—has placed these conditions at the forefront of contemporary biomedical research. NDDs are characterized by the progressive loss of neuronal function, ultimately leading to the degeneration of brain tissue. The most prominent neurodegenerative diseases include Alzheimer’s disease (AD), Parkinson’s disease (PD), Huntington’s disease (HD), and amyotrophic lateral sclerosis (ALS) [1].

AD is a multifactorial, progressive neurodegenerative disorder that arises from complex interactions among genetic, environmental, and lifestyle-related factors [2,3]. Current epidemiological data indicate that AD constitutes a growing public health concern, both in Europe and globally. The global incidence rate increased from 507.96 cases per 100,000 individuals in 1990 to 569.39 cases per 100,000 individuals in 2019 [4]. In Europe, the average prevalence is estimated at 5.05%, with a higher proportion of cases observed in women (7.13%) compared to men (3.31%) [5].

Similar to AD, the global incidence of PD has also increased dramatically. According to 2021 data, approximately 11.77 million individuals worldwide were living with PD. The age-standardized incidence rate was 15.63 per 100,000 people, while the age-standardized prevalence rate was 138.63 per 100,000 people. These data indicate a significant increase in both the incidence and prevalence of the disease over recent decades. The study also highlighted that the burden of PD, as measured by disability-adjusted life years (DALYs), was higher in men than in women. Projections indicate that by 2035, all indicators associated with PD—including incidence, prevalence, and DALYs—will continue to rise, highlighting the increasing impact of the disease on global public health [6].

HD is a rare neurodegenerative disorder, with an estimated global prevalence of 2.7 per 100,000 individuals. It is a progressive condition with a well-defined genetic etiology, and its symptoms are associated with dysfunction in specific brain regions. HD is characterized by a triad of motor, cognitive, and psychiatric disturbances. Symptoms typically manifest between the ages of 35 and 50, although onset can occur at any age, from childhood to late adulthood [1]. The hallmark of HD is the degeneration of neurons in the putamen, caudate nucleus, and cerebral cortex [7]. Brain atrophy, particularly in the striatum, where profound neuronal loss is observed, is also a well-documented feature of HD [8].

ALS is a rare, progressive, and fatal NDD, recognized as the most common form of motor neuron disease in adults. It affects both upper and lower motor neurons, leading to muscle weakness, paralysis, and ultimately death [9]. The global average prevalence of ALS is 4.42 per 100,000 individuals, while the incidence rate stands at 1.59 per 100,000. These rates vary regionally, with the highest figures reported in North America and Europe and the lowest in Asia and South America [10]. The pathogenesis of ALS is multifactorial, involving a range of interconnected mechanisms, including protein aggregation, glutamate excitotoxicity, oxidative stress (OS), mitochondrial dysfunction, apoptosis, and chronic neuroinflammation within the central nervous system (CNS) [11].

Despite the heterogeneity of clinical symptoms—stemming from the degeneration of distinct neuronal cell types and CNS regions—neurodegenerative diseases typically manifest in two major forms: sporadic (nonfamilial) and familial (genetically inherited). These disorders share several core pathological mechanisms, including dysfunction in the autophagic, ubiquitin–proteasome, and lysosomal systems, as well as abnormal protein misfolding and aggregation. Additionally, OS and neuroinflammatory processes are recognized as central contributors to disease progression. In recent decades, extensive epidemiological, clinical, and experimental research has demonstrated that activation of the innate immune system and sterile inflammation within the CNS not only accompany neurodegeneration but also actively drive its progression. As a result, neuroinflammation is now considered a fundamental mechanism underlying the pathophysiology of many NDDs [12].

Neurodegenerative diseases currently remain incurable, and available therapies primarily focus on slowing disease progression, alleviating symptoms, and improving patients’ quality of life, including the treatment of comorbidities. The number of diagnoses has increased significantly over the past decades. This rise is largely due to increased life expectancy—which makes age-related NDDs more common—and advances in early diagnosis, enabling more frequent detection and reporting of these conditions to healthcare systems. As the number of patients continues to grow, there is an increasing need for integrated models of care and a robust health data infrastructure, including the development and implementation of advanced machine learning algorithms to support clinical decision-making [13].

An interesting source of substances that could be used to treat NDDs and alleviate their symptoms is fungi. It is estimated that there are more than 1.5 million species of fungi on Earth, but only about 5% have been studied so far. Despite their enormous diversity, fungi remain one of the least studied groups of organisms. Although approximately 100,000 species have been described to date—and an even smaller number analyzed for their pharmacological properties—fungi have already contributed significantly to the development of effective drugs and fungicides, such as the following:

- Antibiotics: penicillins, cephalosporins, and fusidic acid;
- Antifungal drugs: griseofulvin, strobilurins, and echinocandins;
- Statins (cholesterol-lowering drugs): mevinoline, lowastatin, and simvastatin;
- Immunosuppressive drugs: cyclosporine [14,15].

**Figure 1:**
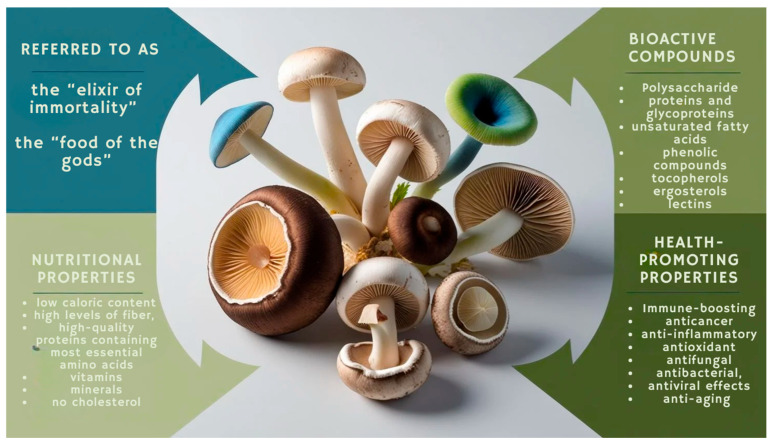
Nutritional properties and bioactive compounds of health-promoting mushrooms.

Moreover, fungi are considered a valuable food source, offering nutritional and health-promoting properties. They are low in calories, high in fiber, and rich in complete proteins, vitamins, and minerals. They also lack cholesterol and serve as a valuable reservoir of biologically active compounds with nutraceutical potential. In addition to their nutritional value, mushrooms have been recognized in folk medicine for thousands of years due to their wide range of medicinal and preventive uses (Figure 1) [16,17,18]. Fungi produce various secondary metabolites, including phenolic compounds, polyketides, terpenes, and steroids. Although these substances do not offer nutritional value, they exhibit significant pharmacological effects, particularly in supporting antioxidant defense mechanisms [19]. This makes fungi a promising resource for the development of novel pharmaceuticals. Currently, mushrooms are gaining increased recognition both as functional foods and as a source of nutraceuticals capable of supporting treatment, alleviating symptoms, and preventing the progression of various diseases [17].

Modern research is shifting from merely confirming the traditional medicinal uses of fungi to identifying and isolating specific active compounds that target particular conditions.

Mushrooms with health-promoting properties can be categorized into several groups based on their biological activity, applications, and content of bioactive compounds (Table 1) [14,15,18,20,21].

According to official data, approximately 2000 species are classified as medicinal mushrooms, with over 600 documented to have therapeutic properties. In light of increasing interest in medically beneficial fungi, it is currently estimated that around 150,000 fungal species exist, of which only about 10% have been thoroughly studied to date [21].

Some of the most commonly cultivated medicinal mushrooms worldwide include *Pleurotus giganteus*, *Ganoderma lucidum*, *Hericium erinaceus*, *Lentinus edodes*, *Trametes versicolor*, *Schizophyllum commune*, *Flammulina velutipes*, *Agaricus bisporus*, *Aspergillus brasiliensis*, *Tricholoma matsutake*, *Auricularia auricula-judae*, *Coprinus comatus*, *Inonotus obliquus*, *Phellinus linteus*, and *Laetiporus sulphureus*. These are used both as dietary supplements and in medicinal applications [21,22]. In contrast to edible mushrooms, medicinal mushrooms are most commonly utilized in biopharmaceutical applications in the form of powders, bulk materials, or liquid extracts [23].

Research on the use of mushroom-derived substances in the treatment of NDDs is steadily increasing. Therefore, this article provides an overview of the pathogenesis of degenerative diseases, such as AD, PD, HD, and ALS, and highlights fungal compounds that may be used to treat or alleviate symptoms associated with these conditions.

## 2. Pathogenesis of Neurodegenerative Diseases

### 2.1. General Definition of the Inflammatory Response in the Central Nervous System

Neuroinflammation refers to the immune response occurring in the CNS in reaction to harmful agents such as infection, ischemia, or trauma [24]. Key features of neuroinflammation include increased cytokine secretion, microglial activation, immune cell migration, and local tissue damage [25]. When the CNS is exposed to noxious stimuli, immune cells residing in the brain—particularly microglia—become activated. Microglial activation is associated with morphological changes and increased expression of receptors that facilitate migration into the region of damage. Microglia protect neurons and regulate physiological processes (e.g., synaptic pruning), but their chronic activation promotes neurodegeneration [26].

Inflammatory mediators such as cytokines, histamine, and reactive oxygen species (ROS) are secreted by microglia, astrocytes, and other immune cells [27]. Astrocytes play a key role in neuroinflammatory processes by regulating the influx of lymphocytes and phagocytes into the CNS. The immune response is linked to OS, as ROS activate pro-inflammatory genes and chronic inflammation enhances their production, creating a vicious cycle of damage. When this balance is disrupted, inflammatory activity intensifies, leading to the release of cytokines, such as IL-1β, IL-6, and TNF-α, as well as harmful neoepitopes. Although neuroinflammation has a protective function (it aims to restore tissue balance), its chronic course has been shown to underlie many non-specific neurological disorders (NDDs). Prolonged neuroinflammation leads to blood–brain barrier (BBB) damage, neuronal injury, and the acceleration of neurodegenerative disease progression [28,29].

### 2.2. Role of Blood–Brain Barrier

The blood–brain barrier is a specialized structure that separates the CNS from the bloodstream and regulates the flow of substances. The main components of the BBB are endothelial cells connected by tight and adhesive junctions, pericytes, astrocytes, neurons, and extracellular matrix elements, which together form the so-called neurovascular unit (NVU) [30]. Under physiological conditions, the BBB selectively permits the passage of small, lipid-soluble molecules while blocking toxins and immune cells [31]. During inflammation, BBB permeability increases, allowing cytokines and immune cells to cross. Neutrophils are the first to infiltrate the CNS, releasing proteolytic enzymes and ROS that exacerbate damage [32]. Once in the brain, monocytes differentiate into M1 (pro-inflammatory) and M2 (anti-inflammatory) macrophages. M1 macrophages secrete IL-1β, TNF-α, and IL-6, intensifying inflammation; M2 macrophages promote BBB repair. T lymphocytes also modulate the immune response—Th1 cells exacerbate inflammation, while Th2 and Treg cells promote neuroprotection [33].

BBB integrity depends on stable cerebral blood flow, which is regulated by the NVU. Pericyte degeneration and chronic inflammation disrupt both cerebral blood flow and barrier function, promoting further damage and neurodegeneration [34]. Research strongly links BBB dysfunction to a range of NDDs. Imaging studies reveal both localized and widespread abnormalities in BBB structure in patients with such conditions. These defects are associated with disruptions in cerebral blood flow, loss of vascular stability, and chronic inflammation in the cerebral vasculature [35]. There is growing interest in therapies aimed at stabilizing the BBB, reducing neuroinflammation and OS, and protecting the brain from aging and disease [30].

### 2.3. Microglia and Astrocytes—Main Cells Involved in the Inflammatory Response in the Central Nervous System

Microglia are the primary cells of innate immunity in the CNS, accounting for about 5–20% of the glial population, depending on the brain region. They are the first to respond to changes in the brain microenvironment, regulate inflammatory responses, shape synaptic networks, and influence neuronal communication [36]. Under physiological conditions, microglia monitor the environment and regulate neuronal functions [37]. In response to pathological stimuli—such as pathogen-associated molecules or tissue damage—microglia become activated and produce pro-inflammatory cytokines (e.g., TNF-α, IL-1β), chemokines, and reactive oxygen and nitrogen species (ROS and RNS). Initially, this response may be protective, but when chronic, it promotes neurotoxicity and contributes to the development of NDDs such as AD and PD [38].

Functionally, microglia are categorized into two phenotypes: M1 and M2. Activated by toll-like receptors (TLRs) and gamma interferon, M1 microglia secrete mediators that exacerbate tissue damage. M2 microglia, induced by cytokines such as IL-4 or IL-10, promote regeneration, secrete neurotrophic factors (e.g., insulin-like growth factor 1), and express anti-inflammatory molecules, such as arginase-1. The transition between these states is crucial for regulating neuroinflammation. Aging, chronic stress, and metabolic disorders can shift microglial activity toward a pro-inflammatory state, accelerating neurodegeneration [37]. Thus, microglia can act as either neuroprotective or neurotoxic agents, depending on their activation state [39].

Astrocytes are abundant glial cells that support brain homeostasis. They maintain ionic balance, capture excess neurotransmitters, and contribute to the formation of the BBB [40,41]. They are also responsible for providing energy to neurons and oligodendrocytes and promoting myelination. In response to brain injury, astrocytes enter a reactive state, characterized by morphological changes and increased expression of glial fibrillary acidic protein. While this initially promotes repair, chronic activation can increase neurotoxicity and disrupt BBB function [40]. Astrocytes can adopt either a neuroprotective (A2) or neurotoxic (A1) phenotype and produce trophic factors that support neuronal survival [42,43]. In a prolonged reactive state, they form glial scars that impede axonal regeneration and contribute to neurodegeneration. For this reason, modulating astrocyte reactivity is being explored as a potential therapeutic strategy, including in AD [44].

Under neuroinflammatory conditions, microglia and astrocytes work closely together. Microglia rapidly activate and clear cellular debris, then stimulate astrocytes, which in turn secrete inflammatory mediators and signal back to microglia, recruiting peripheral immune cells such as macrophages [45]. Activated microglia weaken BBB integrity through the release of pro-inflammatory cytokines, while astrocytes secrete factors (e.g., vascular endothelial growth factor A) that modulate barrier function. Understanding these dynamics is key to developing therapies for neuroinflammatory diseases and NDDs [46].

### 2.4. Role of Inflammatory Mediators

Inflammatory mediators play a critical role in the pathogenesis of NDDs by disrupting neuronal homeostasis. Key among these are the pro-inflammatory cytokines IL-1β, TNF-α, and IL-6. These cytokines are primarily produced by activated microglia and astrocytes in response to injury or infection and have a well-documented role in promoting neuronal damage and synaptic dysfunction. For example, TNF-α induces neuronal apoptosis via cell death receptors, while IL-1β and IL-6 enhance glutamate excitotoxicity and impair synaptic plasticity—effects that are particularly harmful in AD and PD [47,48].

OS, associated with the overproduction of ROS and RNS, is also a significant pathological mechanism. Microglia release these species to eliminate pathogens, but chronic production leads to lipid peroxidation, mitochondrial dysfunction, and neuronal injury [49,50,51].

Chemokines and nitric oxide (NO), synthesized by iNOS, help sustain the inflammatory environment. NO can react with superoxide to form peroxynitrite, a potent neurotoxin. Prostaglandins—particularly PGE2, produced via cyclooxygenase-2 (COX-2) activity—also play a dual role in regulating inflammation and neuronal signaling; however, during chronic inflammation, their overexpression contributes to neurotoxicity [52].

These mediators form a complex interaction network that perpetuates the inflammatory cycle and promotes neurodegeneration. A deeper understanding of these mechanisms may support the development of targeted therapies to modulate glial responses [26].

### 2.5. Specificity of the Inflammatory Response in Selected Neurodegenerative Diseases (NDDs)

**Figure 2:**
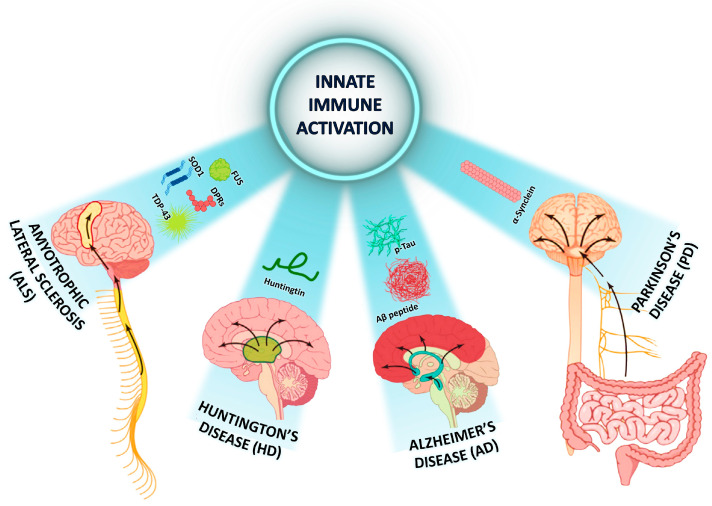
Immune activation and neuropathological hallmarks in common neurodegenerative diseases.

Epidemiological, clinical, and experimental studies confirm that activation of the innate immune system and the resulting sterile inflammation are not only hallmarks of NDDs but also contribute to their progression. Histologically, a common feature of NDDs is the accumulation and proliferation of pathological protein aggregates, either spontaneous or mutation-induced (Figure 2) [12].

The neuroinflammatory immune response plays a key role in the pathogenesis of NDDs. Although brain inflammation is a natural response to damage, in diseases such as AD, PD, and ALS, it becomes chronic and neurotoxic. The nature of this response varies between diseases—it includes differential activation of microglia, astrocytes, and cytokine production. A better understanding of these mechanisms may support the development of targeted therapies [26].

In AD, amyloid beta (Aβ) activates microglia, which migrate to Aβ plaques and attempt to phagocytose them. Although initially effective, this response diminishes over time. In the early stages of AD, the microglial immune response promotes Aβ clearance and can alleviate lesions, as confirmed in animal models [53]. However, when microglial activation becomes prolonged, it contributes to worsening neuropathology. This is likely due to a feedback mechanism referred to as reactive microglia; sustained activation leads to chronic inflammation. This, in turn, results in further Aβ accumulation, and pro-inflammatory cytokine signaling becomes persistent, causing neuronal damage [54]. Ongoing inflammation and recruitment of new microglia and peripheral macrophages further exacerbate Aβ accumulation and synaptic damage. This is accompanied by overproduction of IL-1β, IL-6, TNF-α, and hyperphosphorylation of Tau protein [55,56].

In PD, the neuroinflammatory response involves both microglia and astrocytes, affecting the degeneration of dopaminergic neurons. Disruption of this interaction can lead to neuronal loss and the appearance of motor symptoms characteristic of PD [57]. Chronic microglial activation—triggered by factors such as α-synuclein accumulation, OS, and dopaminergic neuron death—leads to a self-perpetuating inflammatory loop and progressive neuronal damage. In PD, astrocytes undergo astrogliosis. Although initially protective, astrogliosis eventually disrupts glutamate homeostasis and promotes neurodegeneration. PD is also characterized by dysregulation of peripheral immunity, including alterations in T and B lymphocytes, elevated levels of IL-1β, IL-6, TNF-α, and C-reactive protein, as well as the presence of CD4^+^ and CD8^+^ T cells in the *substantia nigra*. Regulatory T cells, which normally suppress immune responses, appear to be reduced or dysfunctional in PD, contributing to an increase in pro-inflammatory Th1 and Th17 lymphocyte subpopulations. At the same time, the reduction in B lymphocytes observed in some PD patients suggests that their dysfunction may contribute to immune imbalance rather than immune overactivity. Overreactive monocytes may cross the weakened BBB, further exacerbating neuronal damage [58,59].

HD is an autosomal dominant neurodegenerative disorder caused by an abnormal expansion of CAG trinucleotide repeats in the *HTT* gene, located on chromosome 4. This mutation leads to protein aggregation, excitotoxicity, mitochondrial dysfunction, and OS [60].

In HD, elevated levels of the translocator protein (TSPO)—a marker of activated microglia—have been observed using positron emission tomography. Increased expression of pro-inflammatory cytokines (IL-6, IL-8, TNF-α), as well as elevated mRNA levels of IL-10, C–C motif chemokine ligand 2, and matrix metalloproteinase 9 in the striatum, have also been demonstrated [61,62,63]. Microglia and astrocytes are major sources of these mediators, and their activity increases with disease progression, suggesting a role in accelerating neurodegeneration. These inflammatory changes may also serve as useful biomarkers and therapeutic targets [64].

ALS is a fatal neurodegenerative disease characterized by the progressive degeneration of upper and lower motor neurons, leading to muscle weakness, paralysis, and death [65]. Although ALS has traditionally been viewed as a disorder confined to motor neurons, growing evidence highlights the critical involvement of CNS immune cells—particularly microglia and astrocytes—in the initiation and progression of the disease [65]. Early observations of inflammatory changes revealed motor neuron loss and aggregates of pathological proteins such as TAR DNA-binding protein 43 and FUS, accompanied by proliferation of activated microglia and astrocytes. Microgliosis and astrogliopathy are consistently observed in postmortem studies and animal models of ALS, along with elevated glial gene expression and reduced neuronal gene expression [66].

Astrocytes, unlike microglia, rarely adopt a neuroprotective phenotype, and their pro-inflammatory activation is influenced, among other factors, by interactions with microglia [67]. Mutations in the *FUS* and *SOD1* genes—including de novo mutations—are among the most common causes of early disease onset, and protein aggregates (TDP-43, FUS) are characteristic of both familial and sporadic forms of ALS. Although peripheral immune cells are present in affected tissues, their role in pathogenesis remains unclear. In contrast, chronic glial activation is considered a key target for potential therapies aimed at modulating the neuroinflammatory response [9].

To facilitate orientation in the key neuroinflammatory mechanisms and to summarize major differences among NDDs, the table below outlines the principal neuroinflammatory and genetic abnormalities characteristic of selected conditions (Table 2).

## 3. Bioactive Compounds in Mushrooms with Health-Promoting Properties and Their Mechanisms of Action on the Inflammatory Response

Nanoparticles derived from mushrooms are gaining increasing significance in NDDs research. Recent studies have confirmed their ability to modulate neuroinflammatory processes and various pathological aspects of these diseases. The primary and secondary metabolite profiles of mushroom species such as *Cordyceps militaris*, *Inonotus obliquus*, *Cyathus africanus*, *Hericium erinaceus*, and *Ganoderma* underscore their therapeutic potential [68,69,70]. Bioactive compounds isolated from these and other mushroom species—including polysaccharides, terpenoids, phenolic compounds, and sterols (Table 3)—exhibit potent anti-inflammatory activity and hold significant therapeutic promise in the treatment of NDDs such as AD, PD, HD, and ALS [71,72,73,74,75].

Chronic inflammation is a key pathological mechanism in these disorders. Studies have confirmed that mushroom-derived compounds inhibit the activation of pro-inflammatory signaling pathways, including nuclear factor kappa-light-chain-enhancer of activated B cells (NF-κB) and the nucleotide-binding oligomerization domain—leucine-rich repeats—and pyrin domain-containing protein 3 (NLRP3) inflammasome. They also reduce the expression of enzymes such as iNOS and COX-2, leading to decreased production of inflammatory cytokines. Additionally, these bioactive components enhance antioxidant and neuroprotective mechanisms, preventing neuronal apoptosis and promoting neurogenesis. As a result, they may slow the progression of neurodegeneration and improve cognitive and motor functions in both in vitro and in vivo models [22,71,96,105,109].

### 3.1. Phenolic Compounds

Phenolic compounds are secondary metabolites that include phenolic acids, flavonoids, lignans, tannins, and stilbenes. Their primary bioactivity lies in their potent antioxidant properties, which involve neutralizing ROS, decomposing peroxides, and inactivating metal ions. This antioxidant capacity helps protect against degenerative conditions, including brain disorders, aging-related processes, and cardiovascular diseases [74,110,111,112,113,114,115].

Vanillic acid, cinnamic acid derivatives, and caffeic acid provide strong protection against hydrogen peroxide. Other acids, such as p-hydroxybenzoic, gallic, and protocatechuic acids, have demonstrated antioxidant, antibacterial, antiviral, antifungal, and anti-inflammatory properties. Additionally, they support gastric juice secretion. Protocatechuic acid, in particular, also exhibits immunomodulatory, spasmolytic, cardioprotective, antithrombotic, and chemopreventive effects. Studies have shown that among edible mushrooms, species such as *Boletus badius*, *Cantharellus cibarius*, and *Pleurotus ostreatus* are richest in phenolic compounds. Depending on the species, they contain between 1 and 6 mg of phenolics and between 0.9 and 3 mg of flavonoids per gram of dry matter. Protocatechuic acid was detected in all tested mushrooms, with the highest levels found in *B. badius*. Other phenolic acids, including p-hydroxybenzoic, sinapic, and cinnamic acids, were detected in varying amounts across different species. Research conducted in Poland and Portugal indicates that wild-growing mushrooms generally contain higher levels of these compounds compared to cultivated varieties. Differences in phenolic composition may result from genetic and environmental factors, as well as storage and sample preparation methods. Overall, edible mushrooms are considered valuable sources of natural antioxidants [72,74,110,111].

The most commonly found flavonoids in mushrooms are myricetin and catechin. The highest levels of phenolic compounds have been recorded in *Boletus edulis* and *Agaricus bisporus*, while *Lactarius deliciosus* exhibited the highest flavonoid content [74].

**Figure 3:**
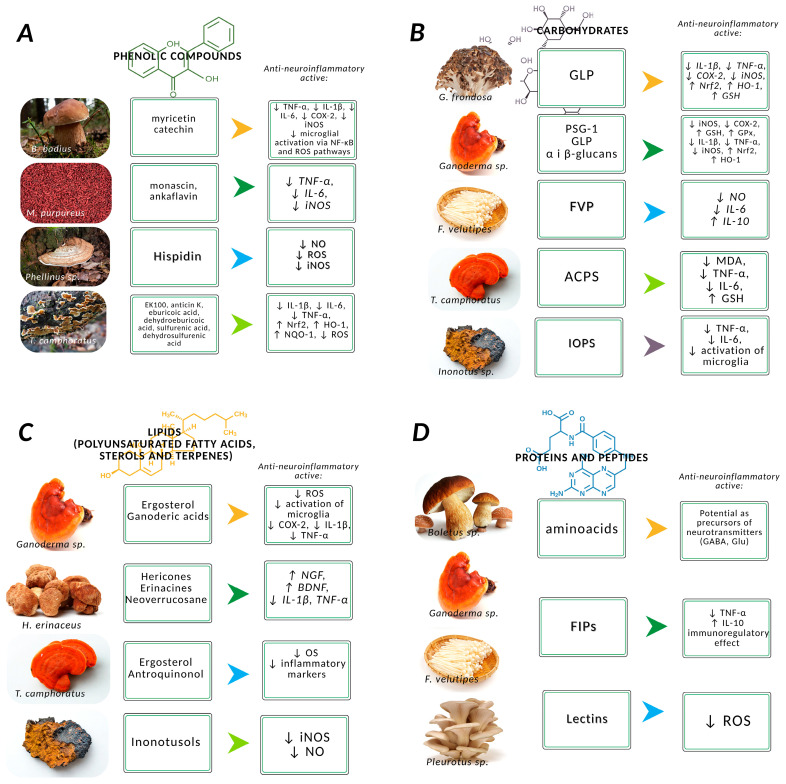
Use of phenolic compounds (**A**), carbohydrates (**B**), lipids (**C**), and proteins and peptides (**D**) derived from mushrooms and characterized by the greatest potential in the treatment of neurodegenerative diseases—protection of neurons from damage caused by oxidative stress and inflammation.

Due to their high phenolic content and antioxidant properties, mushrooms have garnered attention as potential neuroprotective agents. Numerous studies have demonstrated that six phenolic compounds isolated from *Flammulina velutipes* protect PC12 cells against H_2_O_2_-induced OS [17]. Similarly, extracts of *Ganoderma lucidum* rich in phenolic compounds have shown acetylcholinesterase (AChE) inhibitory activity (Figure 3A) [116].

In recent years, additional bioactivities of mushroom-derived phenolics have been documented, including anti-inflammatory, antibacterial, anticancer, and antiviral effects. Compounds derived from *Phellinus baumii* were shown to inhibit NO production in lipopolysaccharide (LPS)-stimulated immune cells, indicating their anti-inflammatory potential. Among these compounds, hispidin—a phenolic metabolite from the *Phellinus* genus—stands out due to its notable therapeutic properties. Hispidin, isolated from cultured *Phellinus linteus*, acts as a non-competitive inhibitor of β-site APP-cleaving enzyme 1 (BACE1), with an IC_50_ of 4.9 × 10^−6^ M. Furthermore, hispidin exhibits strong antioxidant activity by effectively scavenging ROS, thereby contributing to its pronounced anti-inflammatory effects. Additionally, polyphenols from *Inonotus sanghuang* have demonstrated potent antiproliferative and antibacterial activity, further highlighting the diverse bioactive potential of mushroom-derived phenolic compounds [81,113].

Research on *Taiwanofungus camphoratus* (formerly known as *Antrodia cinnamomea*) has identified several compounds with potential anti-inflammatory activity, including six phytosteroids (EK100, antcins K, eburicoic acid, dehydroeburicoic acid, sulphurenic acid, and dehydrosulphurenic acid) and a maleimide derivative, antroquinonol C. These compounds were isolated from both the mycelium and fruiting bodies to evaluate their anti-inflammatory potential. EK100, antroquinonol C, and ergosterol demonstrated the strongest anti-inflammatory effects. In vitro studies revealed that these compounds significantly inhibited NO production in LPS-stimulated BV2 microglial cells, indicating their ability to suppress inflammatory responses. In contrast, antcin K, the eburicoic/dehydroeburicoic acid mixture, and the sulphurenic/dehydrosulphurenic acid mixture did not exhibit similar effects—comparable to the NSAID control, ibuprofen. The most promising compounds—EK100 and antroquinonol C—underwent further in vivo evaluation using the APP/PS1 mouse model of AD. Oral administration of these substances reduced pathological features of neurodegeneration, including amyloid plaque deposition, microglial activation, and behavioral deficits. Both compounds also modulated the nuclear factor erythroid 2–related factor 2/heme oxygenase-1/NAD(P)H:quinone oxidoreductase (Nrf2/HO-1/NQO-1) signaling pathway, supporting their anti-inflammatory and neuroprotective potential. Additionally, EK100 was found to promote neurogenesis. In summary, EK100, antroquinonol C, and ergosterol are the most active anti-inflammatory constituents isolated from *T. camphoratus* and may hold therapeutic potential in treating NDDs associated with chronic inflammation [83].

Monascin and ankaflavin are two yellow pigments produced by *Monascus purpureus* that have attracted scientific interest for their neuroprotective properties. In a mouse model of AD, these compounds demonstrated both anti-inflammatory and antioxidant effects by reducing Tau expression, decreasing beta-amyloid (Aβ) accumulation, and limiting the formation of ROS. They also activated the non-amyloidogenic pathway by increasing the expression of soluble amyloid precursor protein alpha (sAPPα). Despite their potential as effective agents against Aβ-induced AD, safety concerns remain due to the possible production of harmful mycotoxins, such as citrinin, by *M. purpureus*, raising concerns about their applicability in humans [85].

### 3.2. Carbohydrates

Carbohydrates found in mushrooms include simple sugars (glucose, fructose, and galactose), sugar alcohols (mannitol), oligosaccharides (trehalose and melezitose), and polysaccharides. Fungal polysaccharides are long-chain carbohydrate molecules that exhibit considerable structural diversity depending on the mushroom species. They display notable biological activities, including immunostimulatory, anti-inflammatory, antioxidant, antitumor, and gut health-promoting effects. Their bioactivity is closely linked to molecular characteristics such as molecular weight, degree of branching, and monosaccharide composition [16,93]. Both homopolysaccharides (e.g., glucans, chitin, and glycogen) and heteropolysaccharides with complex architectures are present. These include various β-linked glucopyranosyl and galactopyranosyl chains, often branched with sugars such as mannose and fucose. Chitin and β-D-glucans are the primary non-digestible polysaccharides, classified as dietary fiber and structural components of fungal cell walls, especially abundant during fungal development (Figure 3B) [23,74,117,118].

Polysaccharides are among the most abundant and biologically active compounds derived from mushrooms, offering numerous health benefits. Some have been developed and utilized as functional food ingredients, including lentinan from *Lentinus edodes*, schizophyllan from *Schizophyllum* spp., pleuran from *Pleurotus* spp., calocyban from *Calocybe indica*, and ganoderan from *Ganoderma lucidum*. Most of these compounds belong to the group of D-glucans, typically composed of glucose backbones linked by (1→3) and/or (1→6) glycosidic bonds, differing in branching patterns and degrees of substitution [74,117,118].

Fungal polysaccharides represent a promising group of biologically active compounds with a broad pharmacological spectrum and multifunctional actions. They are characterized by low toxicity, a high safety profile, and relatively low production costs, making them attractive candidates for therapeutic applications. These properties have drawn significant scientific interest, and an increasing number of studies highlight their potential to slow the progression of neurodegenerative diseases (NDDs), positioning them as key topics in ongoing research focused on their use as adjunctive or alternative therapeutic agents for such disorders [69]. According to Guo et al. [16], polysaccharides from edible mushrooms possess significant therapeutic potential in clinical medicine, particularly in cancer treatment. Positive clinical trial outcomes for various cancer types underscore the value of these polysaccharides as complementary components to conventional therapies, offering patients additional treatment options and the potential for improved clinical outcomes.

Extraction methods play a critical role in determining the yield, purity, and biological activity of polysaccharides. While traditional methods using hot water or acid/alkali solutions are time-consuming and may degrade the polysaccharides, modern techniques—including ultrasonic-assisted, microwave-assisted, enzymatic, and subcritical water extraction—enhance both extraction efficiency and the biological potency of the resulting products [16].

The neuroprotective effects of fungal polysaccharides include the following:

- Antioxidant activity;
- Anti-amyloidogenic activity;
- Anti-neuroinflammatory effects;
- Anticholinesterase activity;
- Anti-apoptotic effects;
- Anti-neurotoxic effects;
- Anti-ferroptotic effects.

The main pathological processes in neurodegeneration include excessive production of ROS, amyloid aggregation, cholinesterase dysfunction, mitochondrial impairment, and ferroptosis, all of which contribute to chronic inflammation and OS that lead to neuronal damage. Fungal polysaccharides modulate these pathways, enhancing cognitive function, memory, motor performance, and neuronal survival in both in vivo and in vitro models [70].

The antioxidant properties of polysaccharides, particularly prominent in β-glucans and their derivatives, depend on molecular size, hydrogen content, and branching patterns. Polysaccharides derived from *Pleurotus* species (*P. eryngii*, *P. ostreatus*, *P. abalonus*, *P. sajor-caju)* and *G. lucidum* exhibit potent ROS-scavenging and anti-aging effects [16]. Polysaccharides extracted from *P. eryngii* significantly enhanced cell viability, reduced intracellular calcium levels, and mitigated amyloid-induced apoptosis in mouse PC12 cells. These findings suggest the potential of polysaccharides as a therapeutic strategy for delaying the onset and progression of AD [92]. β-Glucans also exert anti-inflammatory effects similar to those of glucocorticoids by inhibiting nitric oxide synthase, COX-2, and pro-inflammatory cytokines, making them promising candidates for the treatment of chronic inflammatory disorders such as AD and PD [16]. Specific examples include β-glucan from *P. ostreatus* alleviating arthritis symptoms in rats and polysaccharides from *Lentinula edodes*, composed of arabinose, rhamnose, and galactose (2.18 kDa), demonstrating anti-inflammatory effects in ulcerative colitis models. Both α-glucans and β-glucans *from L. edodes* modulate immune responses via activation of microRNA (miR-155) and NF-κB signaling pathways in immune cells. An extracellular polysaccharide–peptide complex from *Sanghuangporus lonicericola* was also shown to reduce OS and inflammatory markers (Figure 3B) [16].

In vivo, *F. velutipes* polysaccharides (FVPs) counteracted scopolamine-induced cognitive deficits by reducing OS (preserving antioxidant enzymes and lowering thiobarbituric acid reactive substances), restoring acetylcholine levels through modulation of choline acetyltransferase and AChE activity, and normalizing neurotransmitters such as serotonin, dopamine, and norepinephrine. FVPs also influenced the expression of proteins involved in neurotransmission regulation, including calcium/calmodulin-dependent protein kinase II and connexin 36, and inhibited AChE activity by 18.51% at a concentration of 0.6 mg/mL, highlighting their potential to improve cognitive function [17,70].

Fungal polysaccharides have also been shown to reduce ROS production and lipid peroxidation products while enhancing antioxidant enzyme activities in various models of neurodegenerative diseases. For instance, polysaccharide TLH-3 (4.23 kDa) from *Tricholoma lobayense* exhibited anti-aging effects in D-galactose-treated mice by lowering ROS and malondialdehyde levels. Polysaccharide PSG-1, a high molecular weight compound (1013 kDa) from *Ganoderma atrum*, enhanced antioxidant defenses—such as catalase, superoxide dismutase, glutathione peroxidase, and glutathione—in aging mice induced by D-galactose. Similarly, polysaccharides from *Dictyophora indusiata*, *P. ostreatus*, *Auricularia auricula-judae*, and *F. velutipes* demonstrated neuroprotective properties by decreasing OS markers and boosting antioxidant enzyme levels.

Some polysaccharides act through specific OS-related pathways. ACPS from *Amanita caesarea* has demonstrated significant neuroprotective and anti-Alzheimer’s effects by activating the Nrf2 pathway in HT22 cells and in an AD mouse model. A polysaccharide from *D. indusiata*, rich in various monosaccharides, exerted antioxidant and neuroprotective effects by modulating the same pathway in *C. elegans* models [70].

Research by Han et al. [92] demonstrated that a polysaccharide extracted from *Inonotus obliquus* polysaccharide (IOPS) possesses potent neuroprotective and anti-inflammatory properties in the context of AD. In a cellular model (HT22 cells injured with L-glutamic acid), IOPS increased cell viability, inhibited apoptosis and OS, and restored mitochondrial balance. In an APP/PS1 transgenic mouse model, 8 weeks of IOPS treatment improved memory and cognitive performance, reduced Aβ deposition, and decreased Tau protein hyperphosphorylation in the brain. Moreover, IOPS activated the Nrf2 signaling pathway and upregulated its antioxidant targets, heme oxygenase-1 (HO-1) and superoxide dismutase (SOD-1), underscoring its potential in mitigating OS and inflammation associated with AD [92].

Additionally, *G. lucidum* polysaccharides have been shown to protect against neurodegeneration in conditions such as AD and epilepsy by regulating OS-associated signaling pathways, including extracellular signal-regulated kinase/protein kinase B, c-Jun N-terminal kinase/mitogen-activated protein kinase, and NF-κB [70,93].

A polysaccharide extract from *Grifola frondosa* (GFP) improved escape latency and cognitive performance in APP/PS1 mice at doses of 5–10 mg/kg. It also ameliorated histological damage and necrotic hippocampal neuron morphology, reduced Aβ plaque burden, enhanced microglial and astrocyte activation, and promoted microglia-mediated clearance of pathological amyloid [69]. Similarly, polysaccharides from *G. lucidum* (GLP) modulate the inflammatory response of microglia by promoting the expression of anti-inflammatory cytokines and suppressing pro-inflammatory cytokines. Studies have shown that GLP exerts these effects in BV2 microglial cells as well as in primary microglia activated by LPS or Aβ. Furthermore, GLP modulates microglial morphology, migration, and phagocytosis in the brains of *Danio rerio* (zebrafish), suggesting that its neuroprotective effects are closely related to the regulation of microglial function. Polysaccharides from *T. camphoratus* (APC) have also shown anti-inflammatory effects in mouse models of 6-hydroxydopamine-induced PD. APC treatment alleviated motor symptoms by downregulating NLRP3 inflammasome expression and its pro-inflammatory mediators. Other studies indicate that APC provides neuroprotection by inhibiting ROS–NLRP3 inflammatory signaling pathways in both cellular and animal models of PD. Although the structural characteristics of this polysaccharide are not yet fully elucidated, these findings underscore its therapeutic potential for treating neurodegenerative diseases [70].

### 3.3. Proteins and Peptides

Proteins derived from macrofungi exhibit a broad range of pharmaceutical properties, including immunomodulatory, anticancer, antiviral, antibacterial, and antifungal activities. These bioactive proteins—such as lectins, fungal immunomodulatory proteins (FIPs), ribosome-inactivating proteins, antimicrobial proteins, and ribonucleases—are of considerable therapeutic importance (Figure 3D). Furthermore, studies have shown that dried mushrooms contain a high protein content, ranging from 228 to 249 g/kg of dry matter, which is significantly higher than that of many conventional protein sources. Mushroom-derived proteins and peptides also serve as important bioactive nutrients, supporting digestion and nutrient absorption, modulating immune responses, and inhibiting the activity of specific enzymes [93]. Research indicates that fungal proteins possess a complete amino acid profile, and their nutritional value may even surpass that of animal-derived proteins. They contain numerous essential amino acids as well as significant amounts of glutamic acid, aspartic acid, and arginine. In *Trametes versicolor*, the presence of 18 different amino acids has been identified, including threonine, serine, glycine, and leucine. In addition, ornithine and the non-proteinogenic neurotransmitter GABA have been detected—both known for their beneficial physiological effects [23].

In recent years, numerous bioactive proteins and peptides have been identified in fungi, with lectins and FIPs representing the most abundant groups. Most of these compounds have been isolated from medicinal and rare mushrooms. They primarily exhibit antitumor, antiviral, anti-inflammatory, and immunomodulatory properties, making them promising candidates for therapies aimed at inhibiting tumor growth and metastasis [119,120,121].

Protein hydrolysates of *Pleurotus geesteranus*, prepared using various enzymes, demonstrated that the alcalase hydrolysate exhibited the strongest antioxidant and neuroprotective effects in H_2_O_2_-injured PC12 cells. This hydrolysate functioned by reducing ROS and stimulating antioxidant enzymes. Hydrolysates with higher contents of hydrophobic amino acids also protected the cells by reducing ROS production [96].

Lectins are polysaccharide–protein or polysaccharide–peptide complexes with broad, health-promoting effects, found in the fruiting bodies of fungi. They exhibit anticancer, immunomodulatory, and antiviral activities. Lectins vary in molecular weight, number of subunits, and the types of bound carbohydrates—even within the same fungal species. They have been identified in different parts of the fruiting body—cap, stipe, and mycelium—and their activity depends on the age of the mushroom and the season. For example, a lectin isolated from *Boletus edulis*, which specifically binds to xylose and melibiose, demonstrates mitogenic activity and inhibits HIV-1 reverse transcriptase, suggesting potential applications in preventing immunosuppression in patients undergoing chemotherapy or radiotherapy, or in individuals with AIDS. Despite the wide diversity of fungal lectins, knowledge of their functions and biological activities remains limited [72].

### 3.4. Lipids

The fat content in medicinal mushrooms, such as *Cordyceps sinensis* and *Lentinula edodes* (shiitake), ranges from 0.1 to 5.9 g/100 g and is species-dependent. Mushrooms are rich in unsaturated fatty acids (52–87%), primarily oleic acid (C18:1) and linoleic acid (C18:2), both of which have been associated with anticancer properties. The fatty acid profile may vary depending on the mushroom’s origin—wild specimens typically contain higher levels of polyunsaturated fatty acids than cultivated ones. Shiitake mushrooms also contain various hydroxylated long-chain fatty acids. Unsaturated fatty acids from mushrooms may support cardiovascular health and exhibit antitumor activity [23]. Polyunsaturated fatty acids help reduce blood cholesterol levels. Notably, harmful trans-fatty acid isomers have not been detected in mushrooms. Ergosterol, the primary sterol in fungi, demonstrates strong antioxidant properties, and a sterol-rich diet may aid in the prevention of cardiovascular diseases [74,122,123,124]. Results from Sun et al. [82] showed that ergosterol isolated from *T. camphoratus* significantly reduced LPS-induced proinflammatory cytokines in BV2 and HMC3 microglial cells, possibly by inhibiting the NF-κB, AKT, and MAPK signalling pathways. This may reduce the expression of the microglia activation marker IBA-1 and mitigate LPS-induced neuronal damage. These findings highlight ergosterol’s potential as an effective anti-inflammatory compound [83,103].

Mushrooms also contain tocopherols—natural antioxidants that neutralize free radicals and offer protection against degenerative diseases, infections, and cardiovascular conditions. Linoleic acid, another important compound found in mushrooms, plays a role in anti-inflammatory processes and may reduce the risk of AD by inhibiting AChE and butyrylcholinesterase as well as by suppressing the production of proinflammatory cytokines (Figure 3C) [20,125,126].

Extracts from Anatolian mushrooms such as *Ramaria flava* have demonstrated strong antioxidant activity and inhibition of enzymes related to AD, including AChE and BChE. Additionally, unique cerebrosides isolated from *Albuminosus termitomyces* were shown to stimulate neuronal differentiation, with their activity dependent on the length of fatty acid chains and the presence of hydroxyl groups—highlighting the importance of their structure in exerting neuroprotective effects. In summary, fatty acids and their derivatives from various mushroom species play a key role in suppressing neuroinflammatory processes and supporting neuronal protection and regeneration, indicating their potential in the treatment of NDDs [96].

Extracts from *Cordyceps militaris* have exhibited potent antioxidant properties that may confer protection against degenerative diseases, cancer, and cardiovascular disorders. Lipid components—including fatty acids, esters, and sterols—isolated from ethyl acetate extracts significantly reduced NO production in BV2 microglial cells by 85% at a concentration of 10 μg/mL. This effect was mediated through activation of the Nrf2 and NF-κB pathways, leading to downregulation of proinflammatory genes (iNOS, COX-2) and upregulation of anti-inflammatory genes (HO-1, NQO-1), suggesting potential therapeutic relevance for neurodegenerative conditions [93].

Terpenes are a group of compounds with the general formula (C_5_H_8_)ₙ, while terpenoids are terpenes that contain additional functional groups (-OH, -CHO, =CO, -COOH, -O-O-). Among the most biologically active terpenoid metabolites are triterpenes, which are primarily synthesized by *G. lucidum* and *I. obliquus*. The literature reports high concentrations of these compounds in reishi and chaga mushrooms [23].

Mushroom-derived terpenes constitute a group of volatile, unsaturated hydrocarbons, including monoterpenoids, sesquiterpenoids, diterpenoids, and triterpenoids. Identified sesquiterpenoids include aristolanes, bisabolanes, cuparenes, drimanes, and spiro compounds. Diterpenoids occur primarily as cyathane-type compounds, whereas most triterpenoids isolated from fungi are of the lanostane type. Fungal terpenes exhibit a wide range of health benefits, including antioxidant, antiviral, anticancer, anti-inflammatory, antimalarial, and acetylcholinesterase-inhibiting activities [127,128,129]. Terpenes from *Pleurotus* species, including five monoterpenoids and two sesquiterpenoids from *Pleurotus cornucopiae*, have demonstrated potent anti-inflammatory effects [20,129,130].

Labdane diterpenes (antroquinonols) isolated from the fruiting bodies of *T. camphoratus* (formerly *Antrodia camphorata*) show neuroprotective activity in vitro by protecting PC12 neuronal cells from apoptosis and inhibiting Aβ accumulation. Comparative studies revealed that the fruiting body of *T. camphoratus* is more effective than its mycelium in reducing Aβ40-induced neurotoxicity, limiting Aβ40 accumulation in the brain, and suppressing hyperphosphorylated Tau expression in both in vitro and in vivo models. Fruiting body extracts significantly improved working memory in rat models of AD [85].

Neurotrophic factors are crucial for the proper functioning of neurons, suggesting that substances with similar effects or those that induce their production may be useful in the treatment of neurodegenerative diseases. Extracts from *H. erinaceus* have demonstrated neurotrophic activity and enhanced the process of myelination. A clinical trial involving oral administration of *H. erinaceus* confirmed a significant improvement in cognitive function. Compounds such as hericenones and erinacines, found in *H. erinaceus*, can readily cross the BBB. Erinacines are particularly promising for the treatment of degenerative neuronal disorders and the promotion of peripheral nerve regeneration, as they increase levels of nerve growth factor (NGF). Other yet unidentified bioactive compounds from *H. erinaceus* may also stimulate NGF synthesis. Additionally, dilinoleoyl-phosphatidylethanolamine and 3-hydroxyhericenone F derived from *H. erinaceus* protect neuronal cells against endoplasmic reticulum stress-induced death, a key factor in neurodegenerative diseases [131].

In a study by Wei et al. [107], two novel diterpenoids—16-carboxy-13-epi-neoverrucosan and erinacine L—were isolated from the metabolites produced by *H. erinaceus* cultured on rice medium, along with eleven other compounds, including three cyathane-type diterpenoids, four lanostane-type triterpenoids, three cyclic dipeptides, and one phenol analogue previously known from the literature. The first newly identified compound represents a verrucosan-type diterpenoid derived from basidiomycetes, while the second features a rare hemiketal group. Both compounds were evaluated for their neurotrophic and anti-inflammatory activity. They inhibited NO production in LPS-stimulated BV-2 murine microglial cells. Moreover, compound 1 enhanced NGF-dependent differentiation of PC12 cells at concentrations below 10 μM. Molecular docking studies suggest that both compounds inhibit iNOS activity. The unusual hemiketal structure of compound 2 is likely responsible for its dual neurotrophic and anti-inflammatory effects [107].

Cyathane diterpenoids, including scabronines and sarcodonins, isolated from the fruiting bodies of *Sarcodon scabrosus*, have demonstrated neurite outgrowth-promoting activity. For example, sarcodonin G exerts neuroprotective effects via activation of the TrkB receptor within the tropomyosin receptor kinase (Trk) signaling pathway [107]. Scabronine M, however, was found to inhibit NGF-induced neurite outgrowth, while cyrneines A and B from *Sarcodon cyrneus* promoted neurite extension. Additionally, four novel benzofuran derivatives—ribisines A, B, C, and D—isolated from *Phellinus ribis* extract also stimulated neurite outgrowth in PC12 cells [14].

Studies by Wei et al. [75] revealed that cyathane diterpenoids isolated from *Cyathus africanus* exhibit promising neuroprotective properties in both in vitro and in vivo models of neurodegenerative diseases. Newly identified polyoxygenated neocyathins K–R, along with three known derivatives, increased the number of neurite-bearing cells in PC12 cells stimulated with NGF (20 ng/mL) at concentrations ranging from 1 to 25 μM, without causing cytotoxicity in either PC12 or BV2 microglial cells. Furthermore, one of the isolated compounds inhibited iNOS activity (IC_50_ = 19.8 μM), as confirmed by molecular docking studies [74]. Other studies [131] have described four additional bioactive cyathane diterpenoids—cyathin I, cyathin O, (12R)-11,14-epoxy-13,14,15-trihydroxycyath-3-ene, and allocyafrine B. All four compounds suppressed iNOS expression in BV2 microglia activated by Aβ_1_–_42_ and LPS, as corroborated by molecular docking data [75].

The sesquiterpenoid afrocytinol A, isolated from *C. africanus*, exhibits neuroprotective activity against Aβ1-42-induced neurotoxicity in primary rat hippocampal neurons. This effect is mediated through the reduction of ROS levels, upregulation of the transcription factor Nrf2, and increased expression of its downstream target, heme oxygenase-1 (HO-1). Moreover, afrocytinol A significantly improved neuronal morphology under neurotoxic conditions. These findings highlight the potential of this class of compounds as promising candidates for the development of neuroprotective drugs [69].

Another compound, cyathokorin B, a cyathane diterpenoid isolated from a liquid culture of *Cyathus hookeri*, significantly inhibited NO production in LPS-activated BV-2 microglial cells, suggesting its potential to attenuate neuroinflammation associated with AD [132,133].

Lanostane-type triterpenoids derived from *Ganoderma* species have been extensively studied for their anti-inflammatory and neuroprotective properties. Many of these compounds, including ganoderic acids A, B, D, DM, and H, have been shown to reduce levels of proinflammatory cytokines (IL-1β, IL-6, TNF-α), inhibit microglial activation, and ameliorate cognitive deficits in murine models of AD. For example, ganoderic acid A protects HT-22 hippocampal neurons from OS by lowering ROS levels and regulating apoptosis-related gene expression, while ganoderic acid DM inhibits the expression of inflammatory molecules and microglial activation in in vitro neuroinflammatory models. Ganoderic acid H improves cognitive function and reduces Aβ pathology in APP/PS1 mice. Additionally, compounds such as methyl ganoderate A acetonide and n-butyl ganodionate H effectively inhibit AChE activity, making them promising candidates for drug development targeting AD and other NDDs. Collectively, these findings underscore the significant therapeutic potential of lanostane-type triterpenoids as anti-inflammatory and neuroprotective agents [20,69,81,134].

In a study by Kou et al. [135], seven novel lanostane-type triterpenes, designated inonotosols H–N, were isolated from the fruiting bodies of *Inonotus obliquus*. These compounds exhibited inhibitory effects on NO production in LPS-stimulated BV-2 microglial cells. Western blot analysis showed that the two most promising compounds, inonotosols I and L, effectively suppressed LPS-induced iNOS expression. Moreover, molecular docking studies revealed strong interactions between inonotosols I and L and the iNOS protein [135]. Recent research has confirmed that these newly identified polyoxygenated lanostanoids strongly inhibit NO production in LPS-stimulated BV2 microglial cells, with IC_50_ values ranging from 2.32 to 9.17 μM. Molecular docking confirmed strong binding affinity for the iNOS enzyme, whose expression was significantly reduced by these compounds, while their effects on COX-2 were minimal [69].

## 4. Future Prospects

Despite abundant evidence supporting the beneficial effects of bioactive substances from fungi, most of the data originate from in vitro or preclinical animal studies. Robust clinical trials confirming the efficacy of these compounds in humans are still lacking. For example, the anti-inflammatory and neuroprotective effects of EK100 and anthrodine C from *T. camphoratus* have been documented in mouse models (APP/PS1), but have yet to be confirmed in clinical trials [83,84]. The clinical application of these formulations still faces significant limitations. Key challenges include appropriate formulation, route of administration, safety in long-term use, and potential integration with conventional therapeutic strategies.

Another significant issue is the lack of standardization in fungal extracts. The chemical composition and biological activity of these compounds can vary substantially depending on the species, growing conditions, part of the fruiting body, developmental stage of the fungus, and extraction method [16,20]. For example, the flavonoid content in *Boletus edulis* and *Agaricus bisporus* can vary by several-fold depending on harvest location and storage conditions [72,74], making it difficult to replicate test results and assess the reliability of fungal preparations as nutraceutical products.

### 4.1. Formulation, Administration and Safety of Bioactive Mushroom Products

The pharmacokinetic properties of many compounds extracted from fungi—such as polysaccharides, triterpenes, and phenols—are limited by their high molecular weight, hydrophilicity, and low bioavailability after oral administration. As a result, modern delivery technologies are being developed, including polymeric nanoparticles, lipid carriers (e.g., liposomes), hydrogel systems, and microcapsules. These systems improve the chemical stability of compounds, enable controlled release, and enhance their ability to cross the BBB [16,69].

Polysaccharides from *Ganoderma lucidum*, such as GLP, have shown potent antioxidant and anti-inflammatory effects in animal models of AD through modulation of the Nrf2 and NF-κB pathways [70]. However, their bioavailability following oral administration in humans remains low due to their high molecular weight and poor permeability across the intestinal barrier and BBB. One promising solution is the use of nanoparticles containing *G. lucidum* polysaccharides, which can improve absorption and enable targeted delivery to the nervous system [16]. In parallel, research is ongoing to optimize chemical modifications of these molecules (e.g., sulfation, acetylation) to enhance their pharmacological properties without increasing toxicity [20].

Although many fungal substances exhibit low toxicity in both in vitro and in vivo models, comprehensive toxicological data on long-term use are lacking. It is also important to consider the potential presence of contaminants (e.g., mycotoxins, heavy metals) in natural products—particularly in cases of uncontrolled cultivation or wild harvesting [23,85]. Toxicological data remain limited; for example, some indole-based fungal compounds, such as the tryptamine found in *Tricholoma equestre*, may interact with monoamine oxidase inhibitors, posing a risk of adverse effects [72].

The strong neuroprotective effects of monascin and ankaflavin pigments have also been demonstrated in studies on *Monascus purpureus* [85]. However, the concurrent presence of citrinin—a mycotoxin with nephrotoxic and potentially carcinogenic properties—significantly limits the clinical potential of this raw material without prior detoxification.

### 4.2. Synergistic Actions and Integration with Conventional Therapies

A growing number of studies suggest that mushroom-derived substances can act synergistically with drugs used to treat neurodegenerative diseases, such as AChE inhibitors, anti-inflammatory agents, and antioxidants. In a mouse model of AD, EK100 and anthrodine C from *T. camphoratus* demonstrated potent anti-inflammatory and neuroprotective effects, with efficacy comparable to—or in some respects exceeding—that of ibuprofen [83,84]. These studies also implicated the Nrf2/HO-1/NQO-1 signaling pathways in the mechanisms of action of these compounds.

β-Glucan polysaccharides from *Pleurotus ostreatus* and *Ganoderma lucidum* have been shown to exhibit glucocorticoid-like effects by suppressing iNOS and COX-2 expression while enhancing antioxidant activity and modulating immune responses [16,93]. This mode of action may enable dose reductions of synthetic drugs, thereby lowering the risk of side effects.

Furthermore, the integration of natural and synthetic therapies necessitates in-depth studies using systems-based approaches—such as proteomics, metabolomics, or in silico modeling—to identify potential pharmacodynamic interactions and optimize therapeutic regimens [22,88]. Personalized therapy, based on molecular phenotyping and the selection of appropriate active ingredients according to a patient’s inflammatory profile or genetic background, also holds significant promise.

## 5. Conclusions

NDDs, such as AD, PD, ALS, or HD, lead to progressive loss of neuronal function, resulting in chronic and irreversible motor, cognitive, or psychiatric disorders. Currently, no effective therapies exist that can fully cure these conditions, highlighting the urgent need for new therapeutic strategies capable of halting or slowing their progression. Mushroom-derived substances, which are rich in a diverse array of bioactive compounds, exhibit multidirectional effects—including neurotrophic, anti-inflammatory, antioxidant, and immunomodulatory activities—and represent a promising area of research. Some of these compounds modulate key cellular pathways involved in neuroprotection and regeneration. Additionally, whole mushroom extracts may act synergistically, supporting their potential use in dietary supplements and neuroprotective formulations. However, only a small fraction of the world’s potentially valuable mushroom species have been studied, underscoring the need for further investigation.

Based on the data presented, several key conclusions can be drawn that emphasize the importance of continued research into the therapeutic potential of fungal compounds for neurodegenerative diseases, written as follows:

- Bioactive compounds derived from mushrooms—particularly polysaccharides, phenols, and terpenoids—exert significant anti-inflammatory and neuroprotective effects, giving them therapeutic potential in the treatment of neurodegenerative and other diseases (e.g., cardiovascular disease, cancer).
- Certain fungal components modulate key molecular targets and signaling pathways (e.g., NGF/TrkA, BDNF/TrkB, iNOS, Nrf2/HO-1), enabling precise regulation of cellular processes.
- Unique functional groups in fungal compounds may underlie their specific mechanisms of action.
- Some fungal metabolites are active at very low concentrations and demonstrate lower toxicity compared to synthetic neuroprotective agents.
- Continued research is essential to identify new bioactive compounds, elucidate their mechanisms of action, and explore their potential for therapeutic application in neurodegenerative disease treatment.

## Figures and Tables

**Table 1:** Classification of fungi according to use, biological activity, and bioactive compound content.

Category	Examples	Key Bioactive Compounds	Main Biological Activities	Applications/Remarks
Edible mushrooms	*Pleurotus ostreatus*, *Lentinula edodes*, *Agaricus bisporus*, *Cantharellus cibarius*	Polysaccharides (β-glucans), proteins, ergosterol, phenolic compounds	Antioxidant, mild immunomodulatory, cholesterol-lowering, prebiotic	Human nutrition, functional foods; safe for regular consumption
Medicinal mushrooms	*Ganoderma lucidum*, *Hericium erinaceus*, *Cordyceps militaris*, *Inonotus obliquus*	Triterpenoids, polysaccharides, peptides (FIPs), nucleosides (cordycepin)	Strong anti-inflammatory, immunomodulatory, neuroprotective, anticancer, hypoglycemic	Nutraceuticals, dietary supplements, drug development; often bitter or inedible as food
Toxic mushrooms	*Amanita phalloides*, *Cortinarius orellanus*, *Gyromitra esculenta*	Amatoxins, orellanine, gyromitrin	Cytotoxic, hepatotoxic, nephrotoxic; some act as neurotoxins	Dangerous to health; no therapeutic application; require strict toxicological monitoring
Industrial/fermentation-related mushrooms	*Monascus purpureus*	Monascin, ankaflavin	Anti-inflammatory, antioxidant, cholesterol-lowering	Used in traditional fermentation (e.g., red yeast rice) subject to quality and safety limits

**Table 2:** Main neuropathological and genetic features in selected neurodegenerative diseases.

Neurodegenerative Disease	Main Neuroinflammatory Abnormalities	Major Genetic Causes
Alzheimer’s disease (AD)	- Activation of microglia and astrocytes around amyloid plaques [35]. - Release of proinflammatory cytokines (IL-1β, TNF-α) [35]. - Impaired phagocytic function of microglia [35].	- Mutations in APP, PSEN1, PSEN2 (familial forms) [48]. - APOE polymorphisms (especially APOE ε4) [27].
Parkinson’s disease (PD)	- Microglial activation in the *Substantia nigra* [26]. - Increased production of reactive oxygen species and proinflammatory cytokines [27,60]. - Chronic neurodegenerative inflammation [26].	- Mutations in SNCA (alpha-synuclein), LRRK2, PARK2, PINK1, DJ-1 (inherited forms) [26,60].
Huntington’s disease (HD)	- Reactive astrocytes and microglia in the striatum [8,65]. - Elevated expression of inflammatory mediators (e.g., IL-6) [8,65]. - Glial neurotrophic dysfunction [8,65].	- Expansion of CAG repeats in the HTT gene (huntingtin) [8,65].
Amyotrophic lateral sclerosis (ALS)	- Microgliopathy and astrogliopathy [66] - Release of neurotoxic cytokines and free radicals [27] - Impaired neurotrophic support by astrocytes [27].	- Mutations in SOD1, C9orf72, TARDBP, FUS (familial and de novo cases) [26,27,66].

**Table 3:** Most important bioactive compounds with potential use in the treatment of chronic inflammation in neurodegenerative diseases such as AD, PD, HD, and ALS.

Group of Compounds	Bioactive Substance	Mushroom Species	Experimental Model	Clinical Potencial	Reference
Phenolic compounds	myricetin	*Boletus badius* *Cantharellus cibarius Pleurotus ostreatus*	n.d.	n.d.	[71,73]
	Catechin	*Boletus badius* *Cantharellus cibarius Pleurotus ostreatus*	n.d.	n.d.	[71,73]
	Hispidin	*Inonotus* sp. *Phellinus* sp. *Gymnopilus spectabilis*	in vitro (BV2, PC12), in vivo (MPTP mice)	AD, PD	[20,72,75,76,77,78,79,80]
	EK100 (Ergostatrien-3β-ol)	*Taiwanofungus camphoratus* (formerly known as *Antrodia cinnamomea)*	in vitro (BV2), in vivo (APP/PS1 mice),	AD	[81,82,83]
	antrodin C	*Taiwanofungus camphoratus*	in vivo (APP/PS1 mice)	AD	[81,82,83]
	monascin	*Monascus purpureus*	in vitro, in vivo (LPS-induced)	Experimental model of inflammation and neurotoxicity	[84,85,86]
	Ankaflavin	*Monascus purpureus*	in vitro, in vivo (LPS-induced)	Experimental model of inflammation and neurotoxicity	[84,85]
Carbohydrates	α i β-glucans	*Ganoderma lucidum* *Pleurotus* sp. *Lentinula edodes*	n.d.	n.d.	[16,72]
	Cordycepin	*Cordyceps militaris*	n.d.	n.d.	[72,87]
	FVP	*Flammulina velutipes*	in vitro (BV2)	neuroinflamation model	[17,68,72]
	TLH-3	*Tricholoma labayense*	in vivo (MPTP mice)	PD	[69,88,89]
	PSG-1	*Ganoderma atrum*	in vivo (MPTP mice)	PD	[69,72]
	ACPS	*Amanita caesarea*	in vivo (AlCl_3_-induced)	AD	[69,72,90]
	IOPS	*Inonotus obliquus*	in vitro (BV2), in vivo (Aβ-induced)	AD	[72,91]
	GLP	*Ganoderma lucidum*	in vitro (BV2), in vivo (Aβ, MPTP)	AD, PD	[69,72,87,92]
	GFP	*Grifola frondosa*	in vitro (PC12)	AD	[68,72,93,94]
	ACP	*Taiwanofungus camphoratus* (formerly known as *Antrodia camphorata)*	n.d.	n.d.	[69,81]
Proteins, Peptides	aminoacids (e.g., threonine, serine, glicine, leucine)	*Trametes versicolor* *Pleorotus geesteranus*	n.d.	n.d.	[23,95]
	Lectins	*Boletus edulis* *Pleurotus eryngii*	n.d.	n.d.	[71,96]
	FIPs	*Ganoderma microsporum* *Ganoderma tsugae* *Ganoderma lucidum* *Flammulina velutipes*	in vitro immunological model	AD, PD	[20,97,98]
Lipids	Cerebrosides	*Termitomyces* sp.	n.d.	n.d.	[95,99,100,101]
	Ergosterol	*Taiwanofungus camphoratus* *Ganoderma lucidum* *Auricularia polytricha*, *Cordyceps militaris*	in vitro (BV2), in vivo	AD, PD	[20,81,82,83,87,102]
	Antroquinonol	*Taiwanofungus camphoratus*	in vivo (APP/PS1),	AD	[81,84,87,103]
	Hericones	*Hericium erinaceus*	in vitro (PC12), in vivo (Aβ, MPTP)	AD, PD	[20,72,104]
	Erinacines	*Hericium erinaceus*	in vitro (PC12), in vivo (Aβ, MPTP)	AD. PD	[20,72,87,104,105,106]
	neoverrucosane	*Hericium erinaceus*	n.d.	n.d.	[106]
	Scarboines	*Sarcodon cyrneus*	n.d.	n.d.	[74]
	Sarcodonins	*Sarcodon cyrneus*	n.d.	n.d.	[74]
	Ribisins	*Phellinus ribis*	n.d.	n.d.	[14]
	Cyafricanins (neocyathins, cyathens, cyathans)	*Cyathus africanus*	n.d.	n.d.	[68,74,107]
	cyahookerrin B	*Cyathus hookeri*	n.d.	n.d.	[108]
	ganoderic acids	*Ganoderma* sp.	n.d.	n.d.	[20,68,72,80]
	inonotusols	*Inonotus obliquus*	in vitro (BV2)	AD, PD	[68]

n.d.—not determined.

## Abbreviations

The following abbreviations are used in this manuscript:

AChE	Acetylcholinesterase
ACPS	Polysaccharides from *Amanita caesarea*
AD	Alzheimer’s disease
ALS	Amyotrophic lateral sclerosis
APC	Polysaccharides from *Tajwanofungus camphoratus* (*Amanita camphorata*)
Aβ	Amyloid-beta
BBB	Blood–brain barrier
CAT	Catalase
CCL2	C-C motif chemokine Ligand 2
CD	Cluster of differentation
CNS	Central nervous system
COX-2	Cyclooxygenase-2
FIPs	Fungal immunomodulatory proteins
FUS	Fused in sarcoma gene
FVP	Polysaccharides from *Flammulina velutipes*
GFP	Polisaccharides from *Grifola frondosa*
GLP	Polisaccharides from *Ganoderma lucidum*
HD	Huntington’s disease
HTT	Huntingtin gene
HO-1	Heme oxygenase-1
IL	Interleukin
iNOS	Inducible nitric oxide synthase
IOPS	*Inonotus obliquus* polysaccharide
LPS	Lipopolysaccharide
MAPK	Mitogen-activated protein kinase
NDDs	Neurodegenerative diseases
NF-κB	Nuclear factor kappa-light-chain-enhancer of activated B cells
NGF	Nerve growth factory
Nrf2	Nuclear factor erythroid 2–related factor 2
NLRP3	Nucleotide-binding oligomerization domain—leucine-rich repeats—and pyrin domain-containing protein 3
NO	Nitric oxide
NQO-1	NAD(P)H:quinone oxidoreductase 1
NVU	Neurovascular unit
OS	Oxidative stress
PD	Parkinson’s disease
PSG-1	Polysaccharide from *Ganoderma atrum*
ROS	Reactive oxygen species
SOD1	Superoxide dismutase 1
TLH-3	Polysaccharide from *Tricholoma lobayense*
